# Can Immune Checkpoint Modulation Redefine Ocular Immunotherapy? Emerging Mechanisms, Challenges, and Translational Opportunities—A Comprehensive Review

**DOI:** 10.1167/iovs.66.14.53

**Published:** 2025-11-21

**Authors:** Kai-Yang Chen, Hoi-Chun Chan, Chi-Ming Chan

**Affiliations:** 1 Department of General Medicine, Chang Gung Memorial Hospital (Linkou branch), Taoyuan, Taiwan; 2 School of Pharmacy, China Medical University, Taichung, Taiwan; 3 Department of Ophthalmology, Cardinal Tien Hospital, New Taipei City, Taiwan; 4 School of Medicine, Fu Jen Catholic University, New Taipei City, Taiwan

**Keywords:** immune checkpoint inhibitors (ICIs), T-cell exhaustion (Tex), uveal melanoma, autoimmune uveitis, ocular immunotherapy, tumor-infiltrating lymphocytes, immune evasion

## Abstract

Immune checkpoint inhibitors have revolutionized cancer immunotherapy, and their application is now expanding to various conditions, including prevalent medical issues such as uveal melanoma, autoimmune uveitis, and surface squamous neoplasms. The unique immunological challenges in these cases arise from the immune-privileged nature of the eye, which employs immune evasion mechanisms that often lead to an advanced disease at diagnosis. Recent breakthroughs in immune checkpoints have identified critical regulatory molecules, such as PD-1, CTLA-4, TIM-3, LAG-3, and CEACAM1, which are pivotal in modulating ocular immune responses. The use of novel immunotherapeutic strategies to block these pathways presents new opportunities to treat ocular and inflammatory eye disorders. T-cell exhaustion (Tex) is a significant hurdle in ocular immunotherapy that results in immune dysfunction because of prolonged antigen exposure. Strategies to counteract Tex, including combination therapies that pair PD-1 inhibitors with co-stimulatory agonists such as OX40 and CD137, have shown promise in preclinical murine models. Additionally, dual blockade targeting TIM-3 alongside PD-1 has been found to enhance T-cell responses. An integrated approach targeting multiple pathways may yield improved therapeutic outcomes in eye diseases characterized by immune dysregulation. However, several challenges remain in optimizing immune checkpoint therapies for ocular conditions. Future research should prioritize dual objectives: (1) developing IL-10/TGF-β–promoting therapies to mitigate immune-related adverse events (e.g., retinal vasculitis) and (2) validating LAG-3 + tumor-infiltrating lymphocytes as biomarkers for predicting immune checkpoint inhibitor response in uveal melanoma. It is essential to explore effective combinations of checkpoint inhibitors, determine the optimal timing for their use, and establish better criteria for patient selection. As research on immune checkpoint therapies progresses, they have the potential to transform the management of ocular diseases, offering innovative treatment options for patients who previously had limited choices.

Recent epidemiological studies have illuminated the global burden and immunological complexity of uveitis, underscoring its significance as the leading cause of preventable blindness. The Colombian Uveitis Multicenter Study (COL-UVEA), encompassing 3404 patients, revealed key demographic patterns: a mean age at diagnosis of 41.1 years, a slight female predominance (54.2%), and infectious etiologies accounting for 39.4% of cases—with toxoplasmosis (25.3%) as the most prevalent infectious agent.^1^ Geographic variations in etiology were highlighted by a Greek study of 6191 cases, where herpetic uveitis (14.87%) and toxoplasmosis (6.6%) dominated infectious causes, whereas sarcoidosis and ankylosing spondylitis were prominent noninfectious drivers.^2^ In the United States, uveitis accounts for 10% to 20% of legal blindness cases, with anterior uveitis representing the most common subtype (60–94 cases per 100,000 annually).^3^ These findings emphasize the dual challenges of infectious and immune-mediated ocular inflammation, particularly in aging populations.

The management of eye diseases remains challenging, especially for ocular tumors and autoimmune conditions.^4^ Uveal melanoma is the most prevalent primary intraocular cancer in adults, whereas retinoblastoma remains a leading cause of blindness in children. Inflammatory eye disorders, such as autoimmune uveitis and corneal graft rejection, also involve central immune dysregulation and can severely impair vision.^5^ Conventional treatment options for ocular malignancies typically include enucleation, radiotherapy, and targeted chemotherapy.^6^ Inflammatory eye diseases are primarily managed with corticosteroids and immunosuppressive drugs, although prolonged use of these treatments can result in adverse effects.^7^

The development of immunotherapy offers new possibilities for treating cancers and immune-related disorders, particularly through the use of immune checkpoint inhibitors (ICIs). ICIs potentiate T-cell activity by targeting inhibitory immune checkpoints such as PD-1 and CTLA-4, enhancing immune responses against tumors and autoimmune diseases.^8^ Although ICIs have demonstrated effectiveness in cancer treatment, research on their application in ophthalmology is still evolving. Preliminary studies have indicated that ICIs may enhance outcomes in uveal melanoma and autoimmune uveitis, but more information is required regarding potential immune-related ocular side effects.^9^ This review synthesizes recent advances in immune checkpoint targeting for ocular diseases, emphasizing the unique immune privilege of the eye and implications for tumor and inflammatory conditions, and discusses emerging therapeutic strategies.

## The Immunological Basis of Eye Diseases

### Ocular Immune Privilege and Lymphatic System

The concept of immune privilege in the eye predominantly applies to its internal compartments—the anterior chamber, vitreous cavity, subretinal space, and neural retina. These regions are shielded from systemic immunity by specialized anatomical barriers including the blood-aqueous barrier and blood-retinal barrier (BRB), creating a unique immunosuppressive microenvironment.^10^^–^^14^ The anterior chamber and vitreous cavity show limited lymphatic drainage (“pauci-lymphatics”), which further restricts immune cell trafficking.^11^^,^^15^ The retina, in particular, is protected by both an inner vascular barrier (tight junctions of retinal endothelial cells and glia limitans) and an outer epithelial barrier (retinal pigment epithelium), making it among the most immune privileged tissues in the body^10^^,^^13^ In contrast, the ocular surface (cornea and conjunctiva) is less immune privileged and more exposed to environmental pathogens.^11^^,^^16^ Therefore immune privilege is a property primarily of the intraocular compartments and neural retina, rather than the entire eye.

**Figure 1.**
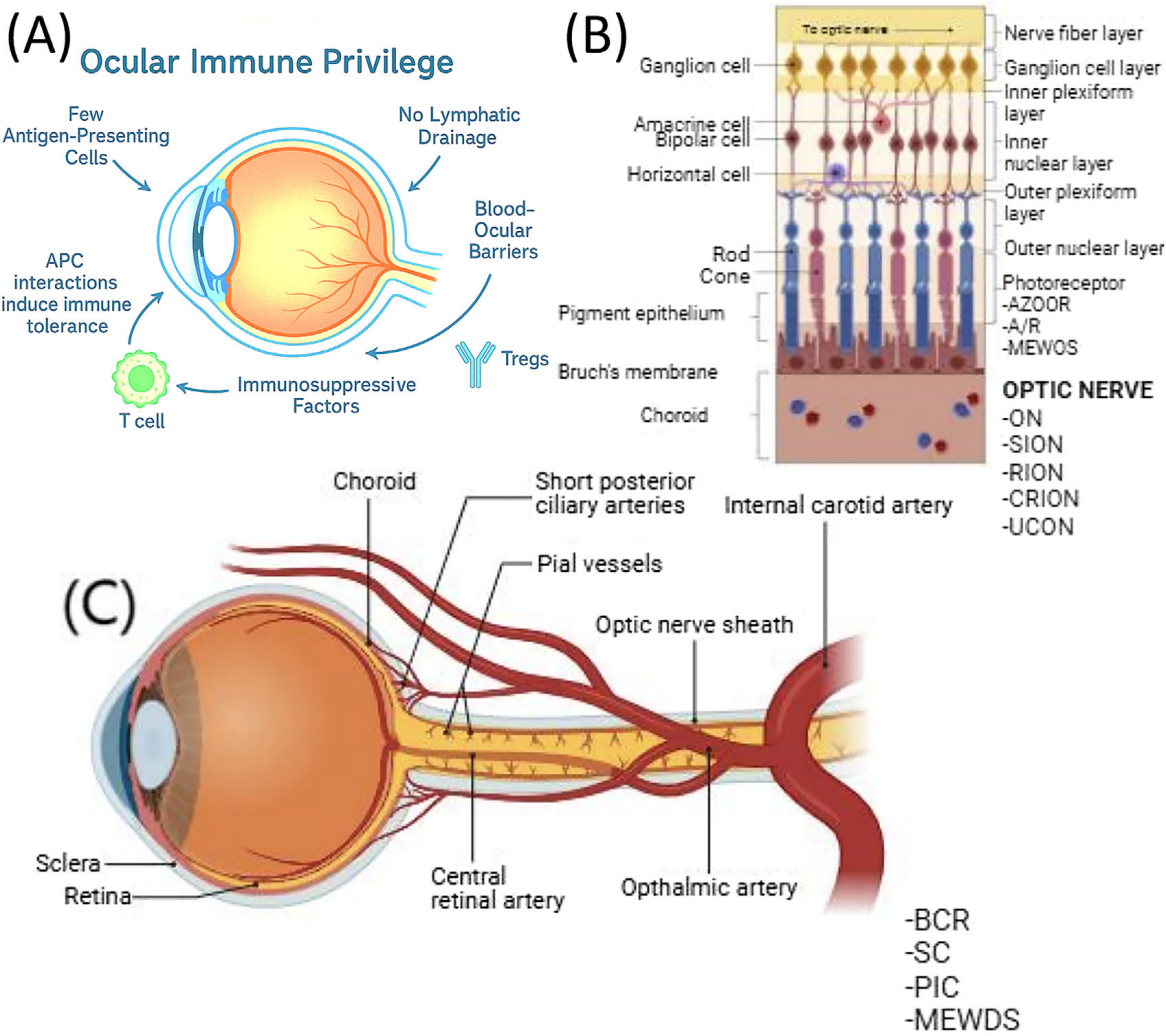
**Anatomical and immunological components of ocular immune privilege.** Ocular immune privilege is maintained through anatomical barriers and immune regulation that protect against excessive inflammation while preserving vision. (**A**) The eye maintains an immune-tolerant microenvironment, known as ocular immune privilege, through multiple mechanisms: paucity of antigen-presenting cells (APCs), absence of lymphatic drainage, presence of blood-ocular barriers (blood-retinal and blood-aqueous barriers), secretion of immunosuppressive factors, and induction of regulatory T cells (Tregs) that suppress immune activation. These mechanisms collectively preserve visual function by limiting inflammation-mediated tissue damage. (**B**) Retinal layers including ganglion, amacrine, bipolar, horizontal, and photoreceptor cells, along with pigment epithelium, Bruch’s membrane, and choroid. (**C**) Cross-sectional view of the globe and optic nerve demonstrating the sclera, retina, choroid, short posterior ciliary arteries, central retinal artery, ophthalmic artery, optic nerve sheath, and pial vessels, highlighting vascular barriers. A/R, acute retinopathy; AZOOR, acute zonal occult outer retinopathy; BCR, blood-cerebrospinal fluid barrier; CRION, chronic relapsing inflammatory optic neuropathy; MEWDS, multiple evanescent white dot syndrome; ON, optic neuritis; PIC, pericytes of inner capillaries; RION, radiation-induced optic neuropathy; SC, scleral canal; SION, secondary ischemic optic neuropathy; UCON, unclassified optic neuropathy.

The anatomical status of immune privilege indicates that ocular immune responses are regulated to prevent damage associated from inflammation (Fig. 1).^17^ Key mechanisms that manage the infiltration of immune cells include the blood-aqueous barrier and BRB, which restrict access to systemic immune responses within the eye.^18^ Historically, it was believed that the eye lacked classical lymphatic drainage. However, recent discoveries have identified lymphatic-like drainage pathways along the ciliary body and Schlemm's canal, challenging the historical view of the eye as being alymphatic.^19^^–^^21^ These pathways facilitate antigen drainage to the deep cervical lymph nodes, enabling peripheral immune education, while minimizing intraocular inflammation.

Regulatory T cells (Tregs) are central to the maintenance of ocular immune tolerance. Ocular pigment epithelial cells induce Foxp3+ Tregs via TGF-β and retinoic acid, suppressing effector T-cell responses and preventing autoimmune damage.^22^ This regulatory network is critical in diseases like autoimmune uveitis, where Treg dysfunction permits unchecked inflammation. Notably, the posterior eye shares lymphatic connections with the central nervous system, creating a unified immune circuit that influences both ocular and neurological immunity.^21^

To address the heterogeneity of uveitis and improve clinical management, the Standardization of Uveitis Nomenclature Working Group established a four-tier anatomical classification system.^23^ This system reveals diverse etiologies: anterior uveitis is predominantly linked to HLA-B27 and spondyloarthritis; intermediate uveitis often associates with multiple sclerosis; posterior uveitis is commonly infectious (e.g., toxoplasmosis) or autoimmune; and panuveitis relates to systemic autoimmune diseases such as Behçet's and Vogt-Koyanagi-Harada syndromes. Epidemiologic data from COL-UVEA show infectious causes account for 39.4% of uveitis, with toxoplasmosis dominating, whereas noninfectious and idiopathic forms comprise significant proportions (Table 1).

**Table 1.** Table Categorizing Uveitis Based on the Primary Site of Inflammation, Prevalence, and Associated Systemic Conditions

Anatomical Classification	Subtypes	Prevalence	Key Associations
Anterior uveitis	Iritis, iridocyclitis	49.5%^1^	Acute presentations linked to HLA-B27 alleles^1^^,^^24^; common in spondyloarthritis
Intermediate uveitis	Pars planitis, posterior cyclitis	5.2%^1^	Multiple sclerosis; idiopathic pars planitis predominates in non-infectious cases
Posterior uveitis	Choroiditis, retinitis	22.9%^1^	Infectious etiologies (e.g., toxoplasmosis: 25.3%); autoimmune retinitis
Panuveitis	Diffuse ocular inflammation	22.3%^1^	Systemic autoimmune conditions (e.g., Behçet's disease, Vogt-Koyanagi-Harada)^25^

Recent epidemiological data from the COL-UVEA of 3404 patients further stratified etiologies:^1^

• Infectious causes accounted for 39.4% of the cases, with toxoplasmosis dominating (25.3%).
• Noninfectious etiologies represented 18.4%, including autoimmune disorders such as HLA-B27-associated anterior uveitis and sarcoidosis.
• Idiopathic uveitis remained prevalent (27.7%), underscoring the diagnostic challenges of ocular inflammation.

This dual anatomical etiological classification enhances diagnostic precision and guides targeted therapies. For example, HLA-B27-associated anterior uveitis often requires systemic immunosuppression, whereas toxoplasmosis-driven posterior uveitis necessitates antiparasitic agents.^1^^,^^24^^,^^25^

Despite these protective mechanisms, immune system activation can still occur in the eye.^26^ For instance, tumor-infiltrating lymphocytes have been observed in uveal melanoma, with immune checkpoint molecules such as PD-1 and CTLA-4 facilitating immune evasion. Similarly, ocular inflammatory conditions, such as uveitis, stem from dysregulated immune responses, where increased permeability of the BRB permits immune cells to infiltrate the intraocular space, leading to chronic inflammation.^27^

### Immune Evasion in Ocular Tumors and Autoimmune Eye Diseases

Uveal melanoma evades immune destruction by upregulating immune checkpoint pathways, particularly the PD-1/PD-L1 axis, which induces T-cell exhaustion and suppresses anti-tumor immunity. This immunosuppressive environment is compounded by the tumor's low mutational burden (∼0.5 mutations/Mb) compared to cutaneous melanoma (15–30 mutations/Mb), limited neoantigen presentation, and distinct PD-L1 expression patterns (nuclear localization vs. membrane-bound), as detailed in Table 2. These factors contribute to the refractoriness of uveal melanoma to checkpoint inhibition, with anti-PD-1 monotherapy achieving only a 3.6% objective response rate compared to 40-50% in cutaneous melanoma. ICIs, including anti-CTLA-4 and anti-PD-1 agents, aim to overcome this evasion by reinvigorating exhausted T cells and have shown improved survival in metastatic uveal melanoma patients, albeit with notable immune-related adverse events such as non-infectious uveitis.^28^^–^^32^

**Table 2.** Comparative Mechanisms of Ocular Versus Cutaneous Melanoma Immune Evasion

Feature	Uveal Melanoma	Cutaneous Melanoma	References
Median mutation burden	0.5 mutations/Mb	15–30 mutations/Mb	31, 34
PD-L1 expression pattern	Nuclear localization (angiogenesis-driven)	Membrane-bound (immune checkpoint)	35
Response to anti-PD-1	3.6% ORR	40%–50% ORR	31

Conversely, autoimmune uveitis arises from dysregulated immune activation, characterized by excessive autoreactive T-cell responses leading to ocular inflammation and tissue damage. In this context, therapeutic strategies promote suppression rather than activation of the immune response. Classical ICIs that potentiate T-cell activity can paradoxically exacerbate or even induce uveitis as an immune-related adverse event during cancer treatment. Instead, immune checkpoint modulators that dampen immune activation—such as CTLA-4-Ig (abatacept)—have demonstrated efficacy in experimental autoimmune uveitis models by expanding Tregs and reducing pro-inflammatory Th17 cytokines, thus curbing inflammation. Therefore, although ICIs have a clear therapeutic role in tumor immunotherapy, their use in autoimmune uveitis remains limited and controversial.^16^^,^^33^

**Figure 2.**
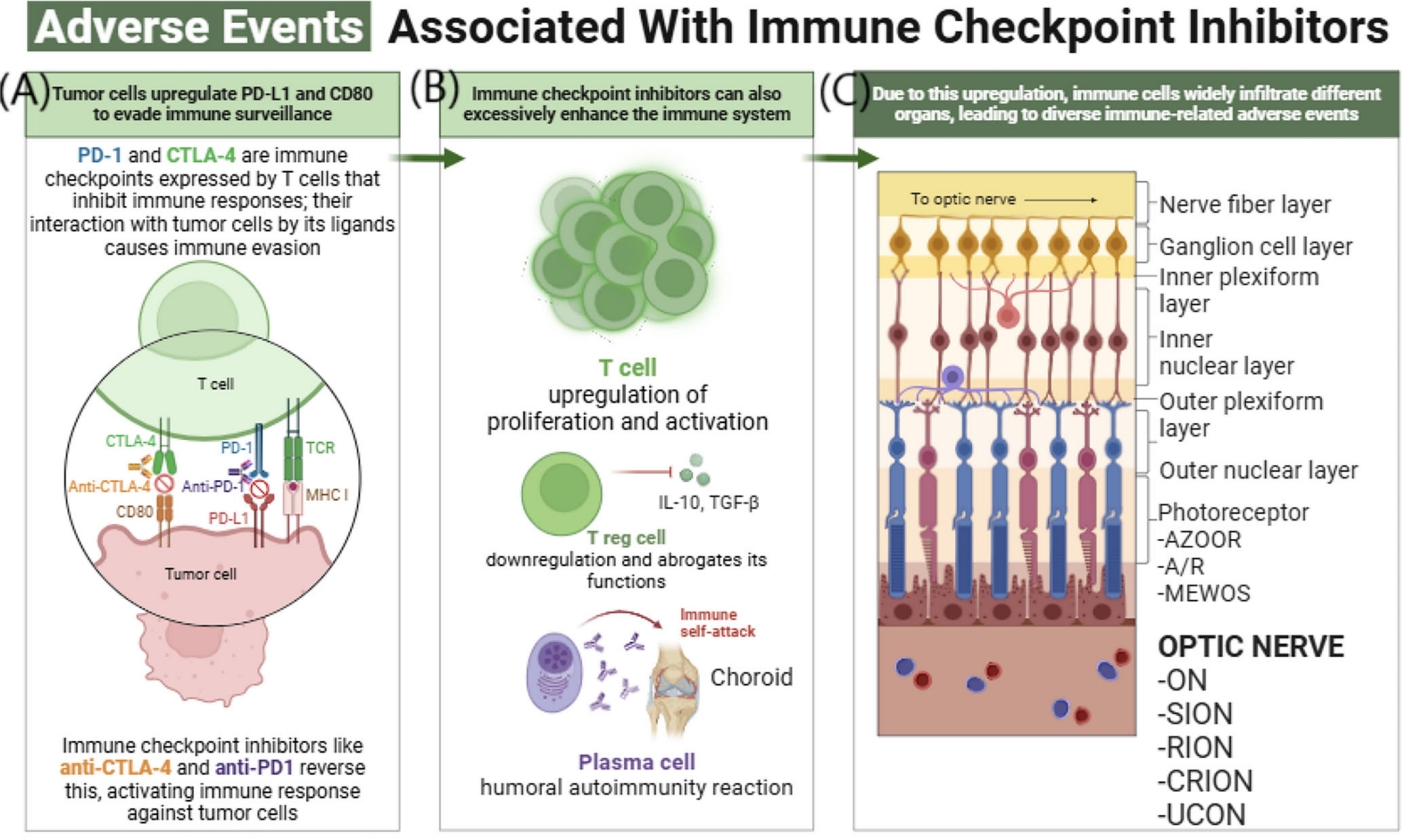
**Mechanisms and ocular adverse events associated with ICIs.** **(A)** Tumor cells upregulate PD-L1 and CD80, engaging immune checkpoints such as PD-1 and CTLA-4 on T cells to evade immune surveillance. ICIs (anti–PD-1, anti–CTLA-4) block these interactions, restoring T cell–mediated antitumor immunity. **(B)** Excessive checkpoint inhibition promotes uncontrolled T cell proliferation and activation, downregulates Treg-mediated suppression, and induces plasma cell–driven autoimmunity, contributing to immune-related adverse events (irAEs). **(C)** Activated immune cells infiltrate retinal layers and the optic nerve, potentially leading to structural injury and ocular complications including retinopathy and optic neuropathies. CD80, cluster of differentiation 80; TCR, T cell receptor; MHC I, major histocompatibility complex Class I.

Autoimmune ocular diseases such as noninfectious uveitis involve the breakdown of tolerance, as explained by the excessive activity of T cells.^34^^–^^36^ Increased concentrations of pro-inflammatory cytokines and abnormal antigen presentation lead to the long-term maintenance of inflammatory conditions and tissue injury. As seen in those mechanisms, immune checkpoint modulation is also involved, presenting possible treatment targets from malignant to inflammatory conditions (Fig. 2).

### Immune Checkpoint Dysregulation in Ocular Pathologies

The ocular microenvironment's immune privilege is subverted in diseases via checkpoint exploitation:

• Uveal Melanoma: Tumor cells upregulate nuclear PD-L1 (nPD-L1), activating STAT3/EGR1 angiogenesis pathways while evading CD8⁺ T-cell surveillance.^37^^,^^38^
• Autoimmune Uveitis: In Behçet's disease, plasma exosomal miR-19b-3p suppresses CD46, skewing Treg/Th17 ratios (1:4.7 vs. 1:1.9 in controls).^39^ CTLA-4 blockade with abatacept reduced relapse rates by 38% in refractory noninfectious uveitis trials.^40^^,^^41^

### Immune Checkpoint Regulation in Ocular Immunity

T cells require both antigen recognition and co-stimulation to achieve full activation during an immune response.^42^ Immune checkpoints play a crucial role in regulating this activation, ensuring that excessive immune responses are curtailed and that normal tissue destruction is minimized. In the context of cancer, tumors can exploit these checkpoints by overexpressing immune checkpoint molecules such as PD-L1, which inhibits T-cell activation and allows the tumor to evade immune destruction.^43^

Immune checkpoint blockade aims to counteract this suppression by using monoclonal antibodies targeting PD-1, PD-L1, or CTLA-4, thereby reactivating T-cell responses against tumors.^44^ These ICIs have shown clinical effectiveness in treating uveal melanoma, and their potential is currently being assessed in inflammatory eye conditions such as uveitis.^45^^,^^46^ Nevertheless, challenges remain, including immune-related adverse events like ocular inflammation and retinal toxicity associated with ICIs, which require further investigation.

## ICIs Under Clinical Investigations for Eye Diseases

**Figure 3.**
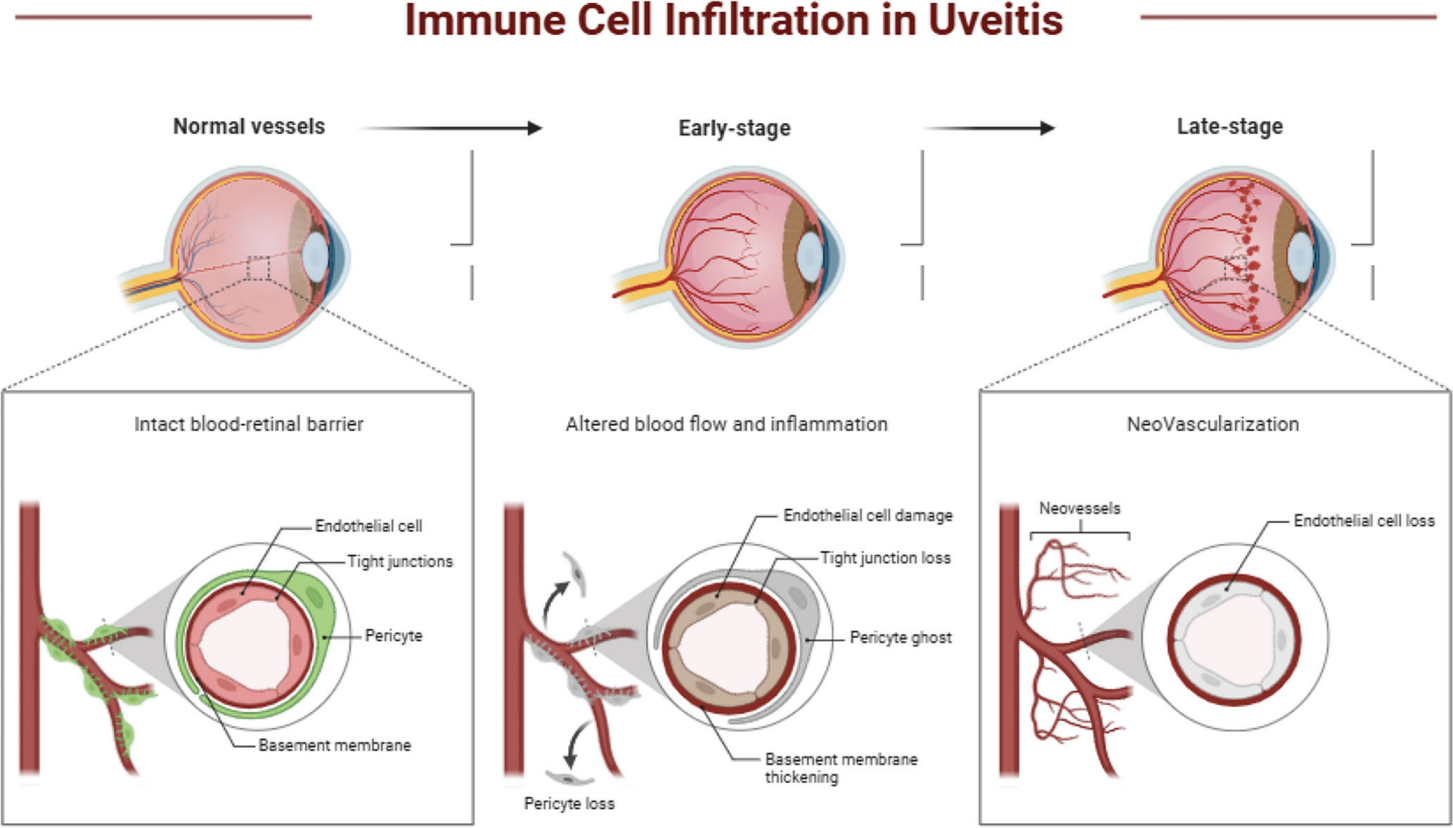
**Immune cell infiltration in uveitis progressively disrupts the blood-retinal barrier.** In normal vessels, an intact blood-retinal barrier is maintained by endothelial cells, tight junctions, pericytes, and the basement membrane. In the early stage of uveitis, altered blood flow and inflammation lead to endothelial cell damage, tight junction loss, pericyte dropout (“pericyte ghosts”), and basement membrane thickening. In the late stage, persistent vascular injury results in endothelial cell loss, contributing to retinal edema and vision impairment.

ICIs have demonstrated promise in modulating immune responses in ocular conditions such as uveal melanoma and autoimmune uveitis. However, most data remain limited to preclinical studies, with clinical trials in ocular diseases yielding modest results so far.^47^ This review aims to clarify the therapeutic potential of ICIs across ocular disorders, emphasizing uveal melanoma, retinal diseases, and autoimmune inflammation. Despite the common occurrence of immune-related adverse events—including uveitis—there is mechanistic rationale for therapeutic benefit in select autoimmune eye diseases. Although ICIs are more commonly associated with causing immune-related adverse events, including uveitis, there is a mechanistic rationale for their potential therapeutic benefit in select cases of autoimmune uveitis. ICIs—such as anti-CTLA-4 and anti-PD-1 antibodies—function by inhibiting inhibitory pathways that suppress T-cell activation, thereby restoring immune activity against aberrant antigens. This immunomodulatory effect can, in principle, be harnessed to rebalance dysregulated T-cell responses that drive chronic ocular inflammation in refractory autoimmune uveitis. Preclinical models have demonstrated that checkpoint blockade—particularly CTLA-4 inhibition—can suppress uveitic inflammation by expanding Treg populations and reducing pathogenic Th17 responses. However, clinical application is currently limited by the risk of exacerbating autoimmune phenomena; thus further studies are needed to identify optimal patient populations and co-therapies that could mitigate these risks^16^^,^^29^ (Fig. 3).

### Immune Checkpoint Inhibitors for Uveal Melanoma

ICIs such as ipilimumab (anti-CTLA-4) and PD-1 inhibitors have demonstrated improved survival outcomes in metastatic uveal melanoma, with ipilimumab increasing two-year survival rates from 12% to 34% compared to chemotherapy.^48^^,^^49^ However, approximately 18% of treated patients develop immune-related adverse events, including noninfectious uveitis, necessitating immunosuppressive management such as steroid-sparing agents like mycophenolate. Clinical trials exploring combination checkpoint blockade, such as LAG-3 and TIM-3 co-inhibition along with PD-1 blockade, are ongoing to enhance anti-tumor efficacy while managing toxicity.^32^

### Immune Checkpoint Modulation in Autoimmune Uveitis

In autoimmune uveitis, the therapeutic goal is to suppress aberrant immune activation rather than to stimulate it. Classical ICIs, by enhancing T-cell responses, are known to exacerbate or trigger uveitic inflammation and are not used therapeutically in this setting. Instead, agents that inhibit co-stimulatory signals—such as the CTLA-4 fusion protein abatacept—have shown promise by expanding Tregs and reducing pathogenic Th17-driven inflammation in experimental models. Future directions may include developing tailored immunomodulatory therapies that balance checkpoint signaling to restore immune homeostasis without provoking autoimmunity.^33^

### Programmed Cell Death Protein 1 (PD-1)/Programmed Death Ligand 1 (PD-L1)

PD-1 is found to be an inhibitory receptor that is present on several immune cells, whereas PD-L1, its principal ligand, can be detected in a large number of tumor tissues. Activation and proliferation of T cells are inhibited when PD-1 and PD-L1 come together so that tumors may have the possibilities of immune evasion.^50^ In uveal melanoma, PD-L1 expression found within the ocular tumor has been linked to poor prognosis because it helps in immune evasion within the tumor microenvironment.^51^ Recent findings propose combining PD-1 inhibitors along with radiation therapy to boost the anti-tumor immune response, thus enhancing the performance of patients with ocular tumors. PD-1 inhibitors have also shown compatibility for use with several other therapies like dendritic cell vaccines and chemotherapy to enhance immune responses and reduce recruitment of Tregs: cells that generally contribute to immune suppression within tumors.^52^

Although showing promising preclinical results, clinical trials of PD-1/PD-L1 inhibitors in eye diseases have been variable. For instance, PD-1 blockade has efficacy in some mouse models but has not been found, with all major clinical trials for tumors in the eye, to improve overall survival or progression-free survival significantly.^53^ A multicenter study of 56 metastatic uveal melanoma patients showed only 3.6% achieved partial responses to pembrolizumab/nivolumab, with median progression-free survival of 2.6 months.^31^ This resistance correlates with nPD-L1's STAT3/EGR1 signaling axis promoting tumor vasculature^35^ and hepatic microenvironment-induced T-cell exhaustion.^34^ Emerging combination strategies (e.g., HDAC inhibitors to block nPD-L1 translocation) may overcome these barriers, as evidenced by RP2 oncolytic virus + nivolumab trials achieving 29.4% response rates via enhanced CD8⁺ infiltration.^35^^,^^54^ Nevertheless, this may create new avenues for ocular tumors and autoimmune uveitis therapy through PD-1/PD-L1 blockade combined with standard therapies.

### Cytotoxic T-Lymphocyte Associated Protein 4 (CTLA-4)

CTLA-4 represents a significant immune checkpoint in ocular conditions, especially those characterized by immune dysregulation, such as autoimmune uveitis. It serves as a negative regulator of T-cell activation, thereby curbing excessive immune responses that could result in autoimmune damage.^55^ Consequently, the use of CTLA-4 blockade in ocular diseases is anticipated to enhance anti-tumor immunity by promoting T-cell activation and diminishing the inhibitory effects of Tregs.^56^

CTLA-4 blockade with ipilimumab improved two-year survival in metastatic uveal melanoma (34% vs. 12% with chemotherapy), but 18% developed non-infectious uveitis requiring steroid-sparing agents like mycophenolate. In contrast, CTLA-4-Ig (abatacept) suppressed experimental autoimmune uveitis by expanding Tregs (CD4⁺CD25⁺Foxp3⁺ cells increased by 2.7-fold) and reducing Th17 cytokines (IL-17A ↓89%).^40^

**Figure 4.**
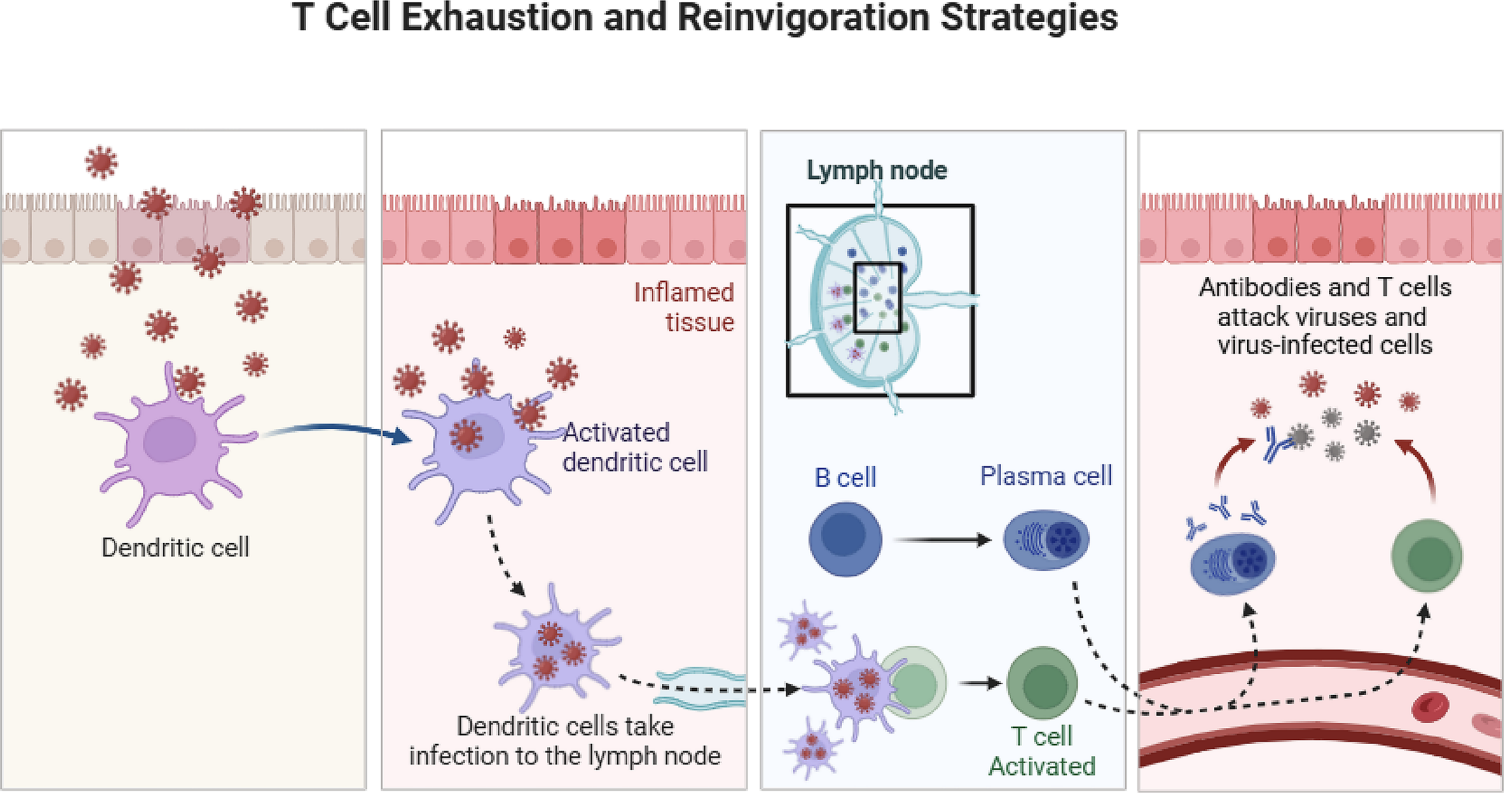
**Mechanism of T-cell activation and reinvigoration during infection.** Dendritic cells recognize pathogens at the site of infection and become activated in inflamed tissue. These antigen-presenting cells migrate to the lymph node, where they stimulate B cells to differentiate into plasma cells producing antibodies, and activate T cells to mount cellular immune responses. The coordinated action of antibodies and cytotoxic T cells eliminates viruses and virus-infected cells, thereby restoring immune function and preventing T-cell exhaustion.

The blockade of CTLA-4 leads to a reduction in T-cell responses against tumors, which can also have implications in autoimmune scenarios. This dual functionality can sometimes result in adverse effects, including ocular inflammation and other immune-related complications.^57^ Therefore additional research is essential to elucidate the various resistance mechanisms and to develop optimal combination strategies for the use of CTLA-4 inhibitors in ocular immunotherapy (Fig. 4).

### T-cell Immunoglobulin and Mucin-Domain Containing-3 (TIM-3)

TIM-3 has emerged as a significant immune checkpoint in the field of ocular immunotherapy. It plays a role in negatively regulating immune responses by facilitating the exhaustion of CD8^+^ T cells and enhancing the activity of Tregs.^58^ Elevated levels of TIM-3 expression have been associated with poorer prognoses and more aggressive forms of ocular tumors and inflammatory diseases. In preclinical studies, the inhibition of TIM-3 has demonstrated the potential to reverse immune suppression and enhance anti-tumor responses. Initial clinical trials exploring the combined inhibition of TIM-3 and PD-1 suggest that targeting both pathways may synergistically enhance immune responses, indicating a promising avenue for therapeutic interventions in ocular conditions.^59^

Although the application of TIM-3 blockade in eye diseases is still in its early stages, a strategic approach that combines TIM-3 and PD-1 inhibition could provide a viable treatment option for both tumors and autoimmune eye disorders.^60^ Ongoing trials are examining this combination in the context of ocular malignancies, with preliminary findings suggesting that this strategy may address some of the challenges associated with single-agent therapies.^61^

### Lymphocyte Activation Gene 3 (LAG-3)

LAG-3 is an immune checkpoint found on various immune cell types, playing a crucial role in suppressing cytokine release and modulating immune responses. It preferentially binds to its ligand MHC-II rather than CD4. LAG-3 contributes to T-cell exhaustion and inhibits immune responses by obstructing T-cell proliferation and activation while simultaneously enhancing suppression mediated by Tregs.^62^ The expression of LAG-3 has been observed in immune cells associated with ocular diseases, particularly in immune dysregulation within tumor microenvironments or inflamed tissues, which contributes to immune evasion.^62^^,^^63^

Preclinical research indicates that targeting LAG-3 may improve immune responses when combined with other immune checkpoint inhibitors such as PD-1 and TIM-3 blockers. In models of ocular diseases, LAG-3 blockade has been shown to stimulate T cell activation and restore immune function, suggesting a promising new therapeutic approach for treating immune-mediated ocular conditions, such as uveitis and ocular melanoma. Clinical trials are currently evaluating the efficacy of anti-LAG-3 monoclonal antibodies in conjunction with PD-1 inhibitors, showing promising potential to enhance immune responses under these conditions.^63^

### Adverse Ocular Events and Insights From ICI

ICIs targeting CTLA-4, PD-1, and PD-L1 have transformed cancer therapy but may trigger immune-related adverse events, including ocular complications. Although uncommon, ocular immune-related adverse events can be vision-threatening and require timely recognition. Reported incidence ranges from 1% to 3% of treated patients, with some cohorts citing higher rates depending on surveillance intensity.^64^^,^^65^ Key ocular manifestations of ICI therapy include uveitis, which occurs in approximately 1% to 2% of patients and may involve any segment of the uveal tract, often necessitating corticosteroid therapy and, in some cases, treatment interruption.^29^

Dry eye and ocular surface disease represent the most frequent events, reported in up to 24% of cases, and although typically mild, they can occasionally present with severe complications.^16^ Neuro-ophthalmic complications such as optic neuritis, cranial neuropathies, and orbital myositis are less common, with an estimated incidence of around 0.5%, but carry a significant risk of profound visual loss.^66^ In addition, rarer entities including autoimmune retinopathy and orbital inflammation have been described in association with multiple ICIs, further underscoring the spectrum of immune-mediated ocular toxicities.^67^ These toxicities reflect disruption of ocular immune privilege, a mechanism essential for preserving vision. Current management relies on corticosteroids and multidisciplinary care, while future strategies may focus on targeted immunomodulation (e.g., cytokine-based approaches) to control inflammation without compromising antitumor efficacy.

## Other Potential ICIs in Ocular Disease Immunotherapy

ICIs have revolutionized cancer therapy and research is now exploring their potential use in ocular diseases, especially those related to autoimmune or tumor-associated eye disorders. Although these inhibitors are primarily administered as standalone treatments, their effectiveness in this context is somewhat restricted, prompting investigations into their combination with other targeted therapies.^61^^,^^62^ However, the introduction of additional treatments raises concerns about the likelihood of adverse effects, including immune-related inflammation that could harm healthy ocular tissues.

### T-Cell Immunoreceptor With Ig and ITIM Domains (TIGIT)

TIGIT serves as an inhibitory receptor in multiple immune cell types, such as T cells and natural killer (NK) cells. Its expression is notably elevated in immune cells in tumor models and inflammatory eye conditions. Inhibiting TIGIT enhances the effectiveness of PD-1 inhibitors, revitalizes T cell activity, and facilitates the recruitment of immunosuppressive Treg cells that play a role in immune regulation.^63^ Consequently, targeting TIGIT alongside PD-1 inhibitors may present a novel strategy for addressing ocular tumors and inflammatory disorders, such as uveitis; however, clinical trials are currently in the preliminary stages.

### B7-H4

B7-H4 is a co-inhibitory molecule found in immune cells that inhibits T-cell activation and facilitates immune evasion. Research indicates that ocular diseases such as uveal melanoma express B7-H4, which correlates with poorer patient outcomes and resistance to standard treatment options, including radiation therapy. In preclinical studies, the inhibition of B7-H4 has demonstrated potential in reversing immune suppression and enhancing therapeutic responses.^68^ Therefore targeting B7-H4 may represent a viable immune checkpoint strategy for the treatment of ocular cancers and various inflammatory eye disorders.

### V-Domain Ig Suppressor of T Cell Activation (VISTA)

VISTA is an immune checkpoint receptor found in tumor and myeloid cells, including microglia, in the central nervous system. It plays a role in diminishing anti-tumor immune responses while promoting the production of immunosuppressive cytokines.^69^ In the context of ocular diseases, targeting VISTA has shown potential in preclinical studies, especially when used in conjunction with PD-1 blockade, to enhance T-cell activation and facilitate tumor clearance. VISTA modulates immune responses in the ocular environment and presents a compelling target for immunotherapy in conditions such as ocular melanoma and uveitis.^68^^,^^69^

### Indoleamine 2,3-Dioxygenase (IDO)

IDO is an enzyme involved in tryptophan catabolism, which is responsible for immune suppression in the TME. IDO degrades tryptophan and thus inhibits T-cell activation and function - paving the way for immune evasion.^69^ IDO activity is implicated in ocular diseases arising from neoplasms or chronic inflammation. Preclinical studies suggest that inhibiting IDO can enhance immune responses and potentiate the efficacy of therapies such as chemotherapy and dendritic cell vaccines. Targeting IDO may therefore represent a promising treatment strategy for both ocular tumors and autoimmune eye disorders.^70^^,^^71^

### Killer-Cell Immunoglobulin-Like Receptors (KIRs)

KIRs are inhibitory receptors that lie on NK cells and tightly control their activation and responses to target cells. In ocular diseases such as ocular tumors, KIRs may restrict the ability of NK cells to identify and kill tumor cells. Inhibition of KIRs may amplify the cytotoxicity of NK cells against tumors, boosting the anti-tumor immune response.^68^^,^^69^^,^^72^ KIR blockade has been shown to enhance NK cell killing of tumor cells in various preclinical studies, indicating that targeting KIRs may prove to be an effective strategy to improve the immune response against tumors in ocular malignancies such as uveal melanoma.

### B and T Lymphocyte Attenuator (BTLA) in Ocular Immunity

BTLA is an immunosuppressive receptor that plays a crucial role in inhibiting T-cell activation and differentiation. Its significance in ocular diseases, especially autoimmune and inflammatory disorders such as uveitis and dry eye disease, is increasingly recognized. Mice with defective BTLA genes demonstrated heightened T-cell activation, making them more susceptible to autoimmune conditions. This receptor may serve as a critical regulator of immune equilibrium within the eye. The upregulation of BTLA is associated with various complications related to immune evasion in tumors, including ocular tumors such as uveal melanoma.^72^ Therefore targeting BTLA expression in the ocular microenvironment could offer innovative immunotherapeutic approaches to restore immune balance in inflammatory eye diseases and enhance anti-tumor responses in ocular cancers.

### Tumor Necrosis Factor Receptor Superfamily, Member 9 (TNFRSF9, CD137, 4-1BB)

CD137 (TNFRSF9, 4-1BB) is a co-stimulatory receptor of the TNF superfamily, expressed primarily on activated T and NK cells, and plays a crucial role in immune regulation. Studies demonstrated upregulated CD137 expression in ocular malignancies, including uveal melanoma, compared to normal tissues.^73^^,^^74^ In autoimmune uveitis, aberrant CD137 signaling may influence T-cell activation and cytokine production.^75^^,^^76^ CD137 signaling enhances T-cell responses, making it a promising therapeutic target for immune modulation in both ocular malignancies and inflammatory conditions.^73^^,^^77^^,^^78^ Combination strategies using CD137 inhibitors with other immune checkpoint inhibitors may improve therapeutic outcomes through enhanced T-cell infiltration into tumor tissues and reduced inflammation in immune-mediated eye diseases.^77^ Notably, preclinical and clinical studies indicated that targeting CD137 can synergize with PD-1 blockade to promote anti-tumor immunity, although toxicity and the risk of immune-related adverse events must be carefully managed.^73^^,^^77^^,^^78^

### Tumor Necrosis Factor Receptor Superfamily, Member 4 (TNFRSF4, OX40/OX40L)

OX40 is another important member of the TNF receptor family that plays a crucial role in T-cell proliferation, survival, and cytokine production. Its interaction with OX40L influences immune responses within the tumor microenvironment and in various inflammatory diseases.^7^^–^^9^ In ocular autoimmune disorders, such as uveitis and scleritis, dysregulated OX40 signaling can lead to sustained T-cell activation and inflammation, causing tissue damage. In contrast, ocular tumors, including uveal melanoma, may exploit OX40/OX40L signaling to evade immune detection.^9^ Preclinical studies targeting OX40 have shown promise in enhancing anti-tumor immunity while also offering a strategy to modulate immune responses in ocular inflammatory conditions. Therefore OX40-based immunotherapies may represent valuable approaches for treating ocular cancers and immune-mediated eye diseases.

### Glucocorticoid-Induced TNFR-Related Protein (GITR)

GITR is primarily found in T cells, particularly Tregs, where it plays a vital role in immune regulation. Tregs are crucial for sustaining immune tolerance within the eye; however, their dysregulation can lead to tumor immune evasion and chronic inflammatory conditions affecting the eye.^17^ In the context of ocular cancers, such as uveal melanoma, research has focused on GITR activation to enhance effector T cell responses while reducing the suppressive influence of Tregs. Furthermore, excessive suppression by Tregs may result in unchecked inflammation in autoimmune eye diseases such as uveitis. Therefore interventions targeting GITR hold promise for enhancing T cell functionality and re-establishing immune equilibrium.^18^ Recent findings suggest that combining GITR-targeted therapies with existing immune checkpoint inhibitors may yield synergistic effects, potentially increasing the efficacy of immunotherapy for ocular tumors and inflammatory eye disorders.

### Carcinoembryonic Antigen-Related Cell Adhesion Molecule 1 (CEACAM1)

CEACAM1 is a cell surface glycoprotein belonging to the immunoglobulin superfamily (Ig). Its role in immune modulation has been linked to a number of diseases, including ocular disorders.^19^ CEACAM1 interacts with almost all types of immune cells to regulate their activity, leading to immune suppression during pathological conditions. CEACAM1 can also bind to the Neisseria Opa protein, preventing T cell activation and reducing its effector functions, including IFN- secretion. Thus the immunosuppressive potential of CEACAM1 resembles that of well-known immune checkpoints, such as PD-1 and CTLA-4.^26^ Recent studies have evaluated the immunotherapeutic potential of anti-CEACAM1 antibodies and have reported promising results in animal models of malignant melanoma and other malignancies. Blocking CEACAM1 could be a new immunotherapy for ocular diseases, such as uveal melanoma, that would restore immune surveillance.^27^ Additionally, targeting CEACAM1 in combination with other immune checkpoint inhibitors may yield superior therapeutic benefits against eye diseases characterized by immune evasion.

## T-Cell Exhaustion in Ocular Diseases

Tex appears to be a threshold that hampers the success of immune checkpoint therapy in multiple ocular diseases such as uveal melanoma, ocular surface squamous neoplasia, and autoimmune uveitis. Co-expression of LAG-3/TIM-3 in 89% of tumor-infiltrating lymphocytes correlates with 8.3-month shorter metastasis-free survival.^79^ Chronic antigenic exposure within the ocular microenvironment is progressive in terms of loss of function by T cells, which reduces the ability to combat disease.^28^^,^^30^ It often co-occurs with the upregulation of inhibitory immune checkpoints, such as PD-1, TIM-3, and LAG-3, which tend to inhibit T cell activation and effector functions.^36^^,^^42^ They act as immune escape mechanisms, providing an environment conducive to uveal melanoma, where exhausted T cells contribute to tumor survival. T-cell dysfunction induced by prolonged immune activation is related to autoimmune uveitis, resulting in chronic tissue damage from inflammation.^53^^,^^55^ It has been suggested that checkpoint inhibitors targeting exhausted T cells could efflux immune responses and form the basis for therapeutic strategies for immune disorders of the eye.

## Reinvigorating Tex in Ocular Immunotherapy

**Figure 5.**
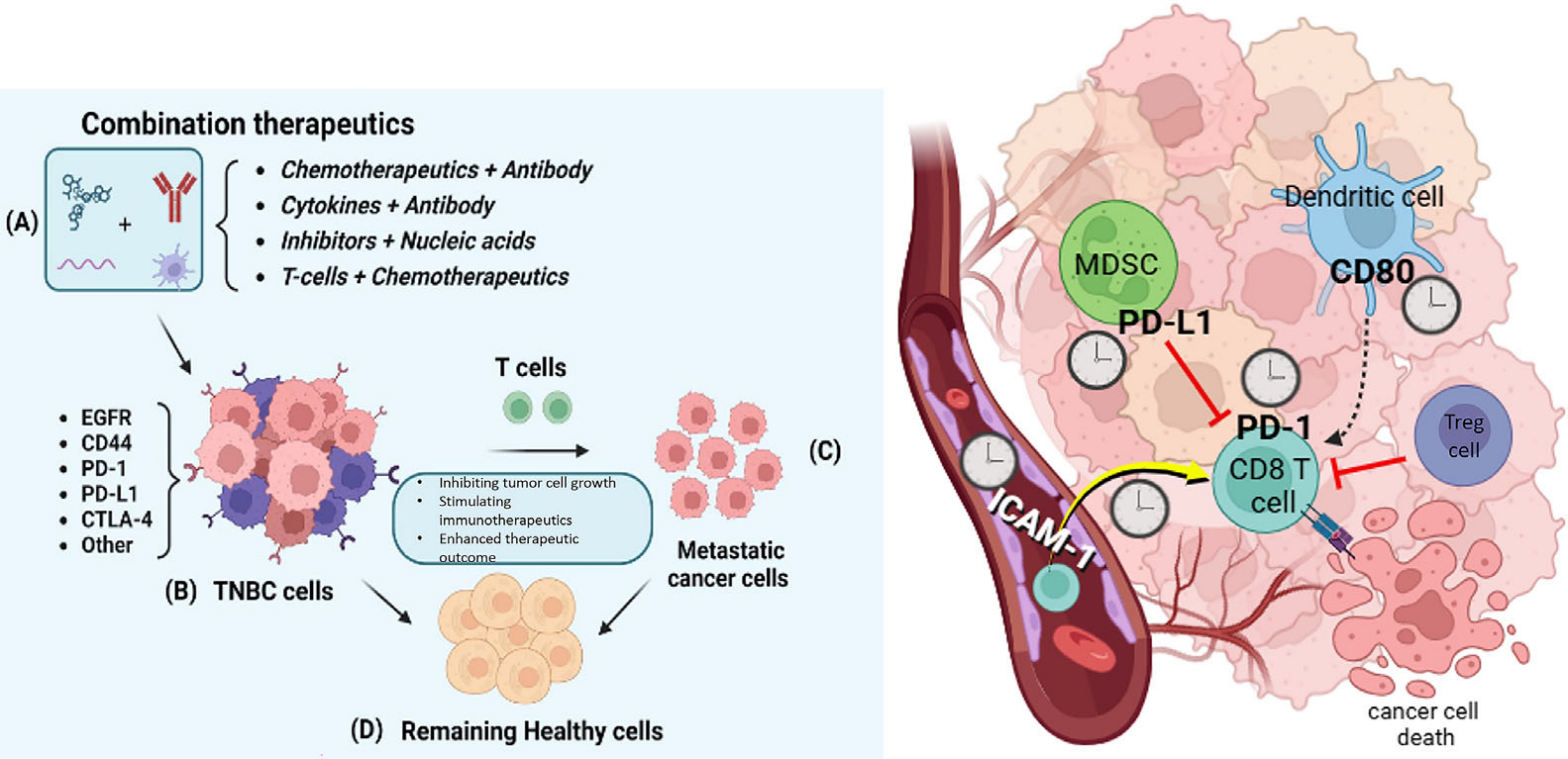
**Combination immunotherapeutic strategies in TNBC.** **(A)** Combination approaches include chemotherapeutics with antibodies, cytokines with antibodies, inhibitors with nucleic acids, and T cells with chemotherapeutics. **(B)** In TNBC cells expressing epidermal growth factor receptor (EGFR), cluster of differentiation 44 (CD44), PD-1, PD-L1, and CTLA-4, these therapies inhibit tumor growth, enhance T cell activity, and improve therapeutic outcomes. **(C)** Enhanced cytotoxic T lymphocyte (CD8⁺ T cell) responses restore immune surveillance and reduce metastatic cancer cells. **(D)** Healthy cells remain largely unaffected. *Right panel*: The tumor microenvironment contains PD-L1–expressing myeloid-derived suppressor cells (MDSCs), Tregs, and dendritic cells (DCs). Blocking PD-1/PD-L1 interactions restores CD8⁺ T cell cytotoxicity, supported by costimulatory signals such as CD80 and adhesion molecules such as intercellular adhesion molecule 1 (ICAM-1), leading to tumor cell death. TNBC, triple-negative breast cancer; CTLA-4, cytotoxic T-lymphocyte–associated protein 4; MDSC, myeloid-derived suppressor cell; DC, dendritic cell; CD80, cluster of differentiation 80; ICAM-1, intercellular adhesion molecule 1; CD8, cluster of differentiation 8.

Restoring T-cell functionality is a critical challenge for the advancement of effective immunotherapy for ocular diseases.^51^^,^^52^ This approach often involves the concept of “removing the brake and pressing the gas pedal” to boost immune system responses. This methodology typically uses ICIs, such as anti-PD-1, to eliminate inhibitory signals (removing the brake) while also enhancing immune activity through co-stimulatory agonists (pressing the gas pedal).^43^ In preclinical studies of uveal melanoma, combination therapies targeting PD-1 along with co-stimulatory pathways, such as OX40 or CD137, have shown potential in reversing T-cell exhaustion. Although TIM-3 expression is elevated in tumor-infiltrating lymphocytes within ocular tumors, it remains a viable target for combination therapies.^44^^,^^47^^,^^50^ Research suggests that the simultaneous blockade of LAG-3 and PD-1 may enhance immune responses and improve treatment outcomes in ocular cancer. As immunotherapy continues to evolve, there is a pressing need for further studies aimed at optimizing ICIs to combat T-cell exhaustion in eye diseases (Fig. 5).

## Conclusions

Immune evasion plays a critical role in the development and progression of ocular diseases, including malignant tumors such as uveal melanoma and autoimmune disorders like uveitis. The unique immune-privileged status of the eye presents both challenges and opportunities for immunotherapy. Although ICIs have transformed cancer treatment, their application in ophthalmology remains largely experimental. Current approaches exploring combinations of ICIs with other therapies are under clinical investigation but have yet to become standard practice. To date, clinical efficacy—particularly in uveal melanoma—has been modest, and overcoming resistance mechanisms remains a formidable challenge. Promising preclinical results using combination strategies, including dual checkpoint blockade targeting LAG-3 and TIM-3 alongside PD-1 inhibition, as well as epigenetic modulation with histone deacetylase inhibitors, offer hope for enhancing anti-tumor immunity. However, further research is essential to optimize these regimens, identify predictive biomarkers for patient selection, refine treatment timing, and mitigate immune-related adverse events. Advances in immune checkpoint modulation hold potential to revolutionize the management of ocular diseases, providing novel therapeutic avenues for patients with currently limited options.
